# Clinical and Biomarker Predictors of Adverse Left Ventricular Remodeling After First STEMI: Insights into Phenotype Variability Using CMR

**DOI:** 10.3390/ph19050794

**Published:** 2026-05-19

**Authors:** Agneta Virbickiene, Vacis Tatarunas, Ieva Ciapiene, Neda Jonaitiene, Justina Jureviciute, Paulius Bucius, Arnoldas Leleika, Ieva Jonauskiene, Liepa Kleizaite, Tomas Lapinskas, Olivija Dobiliene

**Affiliations:** 1 Institute of Cardiology, Lithuanian University of Health Sciences, LT-50161 Kaunas, Lithuania; vacis.tatarunas@lsmu.lt (V.T.); ieva.ciapiene@lsmu.lt (I.C.); 2 Heart Center, Medical Academy, Lithuanian University of Health Sciences, LT-50161 Kaunas, Lithuaniapaulius.bucius@lsmu.lt (P.B.); arnoldas.leleika@stud.lsmu.lt (A.L.); tomas.lapinskas@lsmu.lt (T.L.); olivija.dobiliene@lsmu.lt (O.D.); 3 Faculty of Medicine, Medical Academy, Lithuanian University of Health Sciences, LT-50161 Kaunas, Lithuania

**Keywords:** 20-hydroxyeicosatetraenoic acid, ST-segment elevation myocardial infarction, left ventricular remodeling, cardiac magnetic resonance, biomarkers, eicosanoids

## Abstract

**Background:** Adverse left ventricular remodeling (ALVR) remains an important complication after ST-segment elevation myocardial infarction (STEMI) despite timely reperfusion therapy. Early circulating biomarkers reflecting thromboinflammatory and eicosanoid-related pathways may improve identification of patients at risk of unfavorable remodeling. **Objectives:** To investigate whether platelet count, 20-hydroxyeicosatetraenoic acid (20-HETE), 15(S)-hydroxyeicosatetraenoic acid [15(S)-HETE], and NETosis activity measured on the morning after reperfusion therapy are associated with serial cardiac magnetic resonance (CMR)-defined ALVR after first STEMI. **Methods:** In this prospective single-center study, 93 patients with first STEMI treated with reperfusion therapy, including primary percutaneous coronary intervention (PCI) in 87 patients and thrombolysis followed by PCI underwent baseline CMR at a median of 4 days after PCI and repeat CMR at 6 months. ALVR was defined as a ≥12% increase in both left ventricular end-diastolic volume and left ventricular end-systolic volume at follow-up. Fasting blood samples obtained on the morning after PCI were used to measure platelet count, 20-HETE, 15(S)-HETE, and NETosis activity. Univariable and multivariable logistic regression and receiver operating characteristic analyses were performed. A secondary exploratory analysis evaluated predictors of absolute improvement in left ventricular ejection fraction (LVEF) of ≥10%. **Results:** ALVR occurred in 19 of 93 patients (20.4%). Patients with ALVR had lower platelet count and lower 20-HETE levels at baseline. In the multivariable model, lower platelet count (OR 0.981, 95% CI 0.965–0.996; *p* = 0.015) and lower 20-HETE (OR 0.985, 95% CI 0.970–1.000; *p* = 0.047) were independently associated with ALVR, whereas urea was not significant. In receiver operating characteristic analysis, 20-HETE showed the highest discriminatory ability for ALVR (AUC 0.713, 95% CI 0.594–0.833; *p* < 0.001), followed by platelet count (AUC 0.670, 95% CI 0.546–0.794; *p* = 0.007). By contrast, 15(S)-HETE and NETosis activity were not significant discriminators in the primary analyses. Overall LV function improved during follow-up, with LVEF increasing from 49.0% to 56.0% (*p* < 0.001). In secondary exploratory analysis, higher HDL was independently associated with LVEF improvement of ≥10% (OR 7.84, 95% CI 1.26–48.99; *p* = 0.028). **Conclusions:** Lower platelet count and lower 20-HETE measured on the morning after PCI were independently associated with subsequent CMR-defined ALVR after first STEMI. Platelet count may serve as a simple, clinically accessible marker of risk, while 20-HETE suggests a potential role of eicosanoid-related pathways in remodeling process.

## 1. Introduction

Despite major advances in reperfusion therapy, adverse left ventricular (LV) remodeling remains an important complication after ST-segment elevation myocardial infarction (STEMI) [1]. Even after timely primary percutaneous coronary intervention (PCI), residual myocardial and microvascular injury may promote progressive changes in LV structure and function [1,2]. Because adverse remodeling is strongly associated with systolic dysfunction, heart failure, and unfavorable long-term outcomes, early identification of high-risk patients remains a key clinical priority and an important component of precision cardiovascular medicine [1].

Previous studies of post-STEMI remodeling have primarily focused on infarct size, ischemia–reperfusion injury, and conventional clinical predictors [1,2,3,4]. There is growing interest in incorporating circulating biomarkers that reflect individual biological differences in post-infarction responses. Recent studies have also demonstrated the prognostic value of composite inflammatory markers such as the hemoglobin–albumin–lymphocyte–platelet (HALP) score in STEMI patients [5]. In this context, thromboinflammatory and lipid mediator pathways may offer additional insights into patient-specific mechanisms of myocardial injury and healing. They may also enhance risk stratification beyond traditional markers. Nonetheless, the relationship between thromboinflammatory biomarkers measured early after PCI and serial cardiac magnetic resonance (CMR)-defined LV remodeling after the first STEMI remains poorly understood.

Among clinically relevant candidate markers, platelet count is of particular interest. Platelet count is a clinically accessible biomarker that reflects both thrombotic burden and inflammatory activation in acute myocardial infarction [6,7]. Platelet levels measured early after PCI may capture early dynamic changes in platelet consumption and treatment response. They may also serve as a surrogate for individual variability in thromboinflammatory processes. In addition to platelet count, we evaluated biomarkers reflecting post-infarction inflammation and vascular dysfunction. Among arachidonic acid metabolites, 20-hydroxyeicosatetraenoic acid (20-HETE) is of particular interest because of its vasoactive and pro-inflammatory properties, its association with endothelial dysfunction, and its potential involvement in ischemia–reperfusion injury [8,9,10]. These features suggest that circulating 20-HETE may reflect processes contributing to acute myocardial injury and subsequent ventricular remodeling. We also assessed 15(S)-hydroxyeicosatetraenoic acid [15(S)-HETE], which has been implicated in inflammatory signaling and related lipid mediator pathways involved in myocardial injury and repair [11,12]. However, the relationship between circulating HETE metabolites and serial CMR-defined LV remodeling after the first STEMI remains poorly defined.

Neutrophil extracellular traps (NETs) have also emerged as important mediators of thromboinflammatory injury after STEMI. NETosis-related activity has been associated with larger infarct size, worse ventricular function, and adverse remodeling, suggesting a possible link with post-infarction myocardial injury [13,14]. However, evidence relating NETosis-related activity to myocardial recovery and serial CMR-defined adverse LV remodeling remains limited.

It remains uncertain whether thromboinflammatory and eicosanoid-related biomarkers measured early after PCI are associated with adverse LV remodeling assessed by serial CMR. Their incremental prognostic value beyond established clinical and imaging markers also remains unclear.

In this prospective study, we investigated the association between platelet count measured early after PCI and adverse LV remodeling assessed by serial CMR, and explored circulating 20-HETE, 15(S)-HETE, and NETosis activity as complementary biomarkers reflecting thromboinflammatory and eicosanoid-related pathways. These biomarkers may have implications for individualized risk stratification in precision cardiovascular medicine.

## 2. Results

### 2.1. Patient Characteristics

The final analysis included 93 patients with paired baseline and 6-month follow-up CMR data. The median age was 61 years (55–68), and 72 patients (77%) were male. The most prevalent cardiovascular risk factors were dyslipidemia (85 patients, 91%), hypertension (76 patients, 82%), and active smoking (48 patients, 52%), whereas diabetes mellitus was present in 12 patients (13%). The median body mass index was 28.1 kg/m^2^ (25.0–30.8).

### 2.2. Procedural Characteristics

The median onset-to-balloon time was 345 min (198–533). Median troponin I concentration was 26.6 µg/L (14.2–45.7). Anterior myocardial infarction occurred in 41% of patients. Pre-procedural TIMI 0–1 flow was observed in 72%, whereas TIMI 3 flow after PCI was achieved in 94%. Thrombolysis prior to PCI was performed in 6 patients (6.5%).

### 2.3. Baseline Characteristics According to Adverse Left Ventricular Remodeling

ALVR, defined as a ≥12% increase in both LVEDV and LVESV at follow-up, occurred in 19 patients (20.4%). Baseline characteristics according to ALVR status are presented in Table 1. Patients who developed ALVR more frequently had anterior myocardial infarction (63.2% vs. 35.1%, *p* = 0.027). Differences were also observed in pre-hospital medication use, with higher use of beta-blockers, ACE inhibitors/angiotensin receptor blockers, and calcium channel blockers in the ALVR group. No significant differences were observed in cardiovascular risk factors, blood pressure, or routine laboratory markers, including troponin I, BNP, and creatinine. However, distinct differences in thromboinflammatory and eicosanoid-related biomarkers were observed, suggesting potential variation in biological responses after an infarction among individuals. Platelet count and circulating 20-HETE concentrations were lower in patients who developed ALVR. No significant differences were found for 15(S)-HETE or NETosis activity. On baseline CMR, patients with ALVR had higher LVESV, whereas LVEDV was numerically higher, and LVEF did not differ significantly between groups.

### 2.4. Changes in CMR Parameters from Baseline to 6-Month Follow-Up

Within-patient analysis demonstrated overall improvement in LV function and myocardial deformation over 6 months. LVEF increased significantly, whereas LVEDV remained unchanged. Global myocardial deformation improved, with more negative GLS and GCS values at follow-up, indicating recovery of myocardial function (Table 2).

### 2.5. Factors Associated with Adverse Left Ventricular Remodeling

Univariable and multivariable logistic regression analyses examining factors associated with ALVR are presented in Table 3. In univariable analysis, lower platelet count and higher urea were associated with ALVR, while lower 20-HETE showed a borderline association. Given the limited number of ALVR events, the multivariable model was restricted to three variables to reduce the risk of overfitting. Because 20-HETE was the primary biomarker of interest, 20-HETE, urea, and platelet count were entered simultaneously into the multivariable logistic regression model. In the multivariable model, lower platelet count (OR 0.981, 95% CI 0.965–0.996, *p* = 0.015) and lower 20-HETE (OR 0.985, 95% CI 0.970–1.000, *p* = 0.047) remained independently associated with ALVR, suggesting their potential role as complementary markers of individual thromboinflammatory and vascular response following STEMI. Urea did not reach statistical significance (*p* = 0.051).

### 2.6. Diagnostic Performance of Biomarkers for Adverse Left Ventricular Remodeling

**Figure 1:**
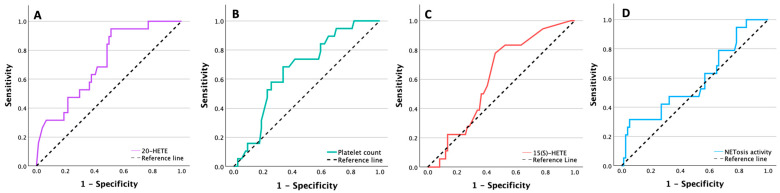
Receiver operating characteristic (ROC) curves for discrimination of adverse left ventricular remodeling (ALVR): (**A**) 20-HETE, (**B**) platelet count, (**C**) 15(S)-HETE, and (**D**) NETosis activity.

Receiver operating characteristic analysis showed that both 20-HETE and platelet count discriminated ALVR with only moderate accuracy (Figure 1, Table 4). Among the tested biomarkers, 20-HETE had the highest AUC (0.713, 95% CI 0.594–0.833, *p* < 0.001), and platelet count showed modest discrimination (AUC 0.670, 95% CI 0.546–0.794, *p* = 0.007). In contrast, 15(S)-HETE and NETosis activity did not significantly discriminate ALVR (*p* = 0.083 and *p* = 0.271, respectively).

Using ROC-derived thresholds, univariable logistic regression identified lower 20-HETE and platelet count, as well as higher 15(S)-HETE, NETosis activity, B-type natriuretic peptide (BNP), and urea, as significant predictors of ALVR (Table 5). In a multivariable model including ROC-derived dichotomized predictors that were significant in univariable analysis (Table 5), lower 20-HETE and platelet count, together with higher BNP and NETosis activity, remained independently associated with ALVR (Table 6). As the ROC-derived cut-offs were defined and tested within the same cohort, the dichotomized analyses shown in Table 5 and Table 6 should be regarded as exploratory, as the reported odds ratios may be affected by optimism and require external validation.

### 2.7. Secondary Exploratory Analysis of Left Ventricular Ejection Fraction Improvement

In a secondary exploratory analysis, factors associated with an absolute improvement in LVEF of ≥10% at 6-month follow-up were evaluated (Supplementary Tables S1 and S2). Greater LVEF improvement was observed in patients with lower baseline LVEF, higher high-density lipoprotein (HDL) cholesterol levels, lower urea levels, and anterior myocardial infarction. In the exploratory logistic regression analysis, higher HDL remained independently associated with LVEF improvement of ≥10% (OR 7.84, 95% CI 1.26–48.99, *p* = 0.028).

### 2.8. Exploratory Correlation Analyses

Exploratory correlations between selected laboratory and biomarker variables and CMR-derived functional, remodeling, and myocardial deformation parameters are presented in Supplementary Tables S3 and S4. Troponin I, alanine aminotransferase, and aspartate aminotransferase showed the most consistent correlations with baseline and follow-up LV function and deformation parameters. Among the study biomarkers, BNP correlated inversely with LVEF; 20-HETE showed a modest association with GLS; and NETosis activity correlated with LV volumes. Given the exploratory nature of these analyses and the absence of adjustment for multiple comparisons, these findings should be interpreted with caution.

## 3. Discussion

This study offers new insights by combining thromboinflammatory and eicosanoid-related biomarkers with serial CMR-defined remodeling outcomes in a prospective STEMI cohort, providing a more individualized view of post-infarction myocardial recovery. In this prospective serial CMR study of first STEMI patients treated with reperfusion therapy, most showed recovery of LV systolic function over 6 months. However, approximately one-fifth developed adverse LV remodeling.

A key finding was that a lower early post-PCI platelet count independently predicted ALVR. Additionally, the link between reduced 20-HETE levels and ALVR indicates a possible involvement of eicosanoid-related pathways in post-infarction remodeling and reveals biologically distinct patient responses. Patients with ALVR more often had anterior myocardial infarction and higher baseline LVESV, emphasizing the importance of initial injury severity in remodeling outcomes. Conversely, 15(S)-HETE and NETosis activity showed no consistent relationship with ALVR. Overall, these results imply that platelet count is the most clinically accessible marker associated with ALVR in this cohort, while the link to 20-HETE is biologically interesting but preliminary.

**Platelet-related mechanisms and inter-individual variability.** Previous studies suggest that platelet-related markers are associated with the severity of myocardial injury after acute myocardial infarction, although these associations vary by marker and timing of measurement. Baseline platelet count has been linked to outcomes after PCI and, in more recent work, to infarct size [6,15]. Platelet reactivity and other platelet-related measures have also been associated with intramyocardial hemorrhage, microvascular obstruction, infarct size, and adverse remodeling [16,17,18,19]. Taken together, these observations support a role for platelet-mediated thromboinflammatory pathways in both acute myocardial injury and subsequent tissue repair. Against this background, our finding that lower platelet count measured on the morning after PCI was associated with later ALVR may reflect increased platelet consumption at sites of coronary and microvascular injury. It may also indicate a more intense inflammatory response during the acute phase of infarction or other nonspecific periprocedural influences. Although this interpretation remains speculative, it is biologically plausible given experimental evidence that platelets participate in post-infarction inflammation and myocardial remodeling [18]. At the same time, platelet count is a nonspecific marker. It may reflect baseline interindividual variation, antithrombotic treatment effects, or hemodilution rather than a direct mechanistic mediator of remodeling. Accordingly, our findings suggest that platelet-related processes may be relevant to later ventricular remodeling, but they do not establish causality.

**20-HETE and eicosanoid pathways in myocardial remodeling.** Beyond platelet count, lower 20-HETE concentration was also associated with subsequent ALVR, although this finding should be interpreted cautiously. 20-HETE is a biologically active arachidonic acid metabolite with complex and context-dependent cardiovascular effects, including regulation of vascular tone, inflammation, oxidative stress, and ischemia–reperfusion injury [9,20]. Experimental studies have generally linked increased 20-HETE activity to greater acute myocardial injury. In contrast, inhibition of 20-HETE-related pathways has been associated with cardioprotective effects in ischemia–reperfusion models [9]. Against this background, the lower 20-HETE levels observed in our patients on the morning after PCI may reflect altered metabolic responses during the acute post-infarction phase rather than a straightforward harmful or protective effect. This inverse association may appear counterintuitive because experimental studies have often linked increased 20-HETE activity with greater ischemia–reperfusion injury, whereas inhibition of 20-HETE-related pathways has been associated with cardioprotective effects. Therefore, lower circulating 20-HETE in our cohort should not be interpreted as direct evidence that reduced 20-HETE promotes adverse remodeling. Instead, the lower 20-HETE levels observed after STEMI may reflect altered synthesis, increased local consumption, redistribution, or degradation of this lipid mediator during the early post-infarction phase. Notably, although 20-HETE differed between groups and showed the highest AUC among the evaluated biomarkers, its association in regression analysis was less robust. This finding suggests sensitivity to model specification and the limited number of ALVR events.

Clinical data specifically linking early post-PCI circulating 20-HETE levels with CMR-defined remodeling after STEMI remain scarce. Accordingly, the present findings should be considered hypothesis-generating. Future studies incorporating serial measurements and integrative lipidomic profiling may better define the role of 20-HETE within broader eicosanoid networks and clarify its potential contribution to risk stratification after STEMI [21].

**Limited role of 15(S)-HETE and NETosis.** In contrast to platelet count and 20-HETE, 15(S)-HETE did not show a consistent association with ALVR in our cohort. It did not significantly discriminate ALVR in ROC analysis and showed no meaningful correlations with baseline or follow-up CMR-derived indices of LV function or remodeling. This may indicate either that 15(S)-HETE is less directly related to CMR-defined remodeling after first STEMI than initially hypothesized. Alternatively, a single early circulating measurement may not adequately capture the relevant lipoxygenase-related processes involved in myocardial healing. Experimental and translational studies suggest that 15-HETE-related pathways may participate in inflammatory and fibrosis-related responses after myocardial injury. However, direct clinical evidence supporting circulating 15(S)-HETE as a predictor of post-STEMI remodeling remains limited. NETosis activity also did not significantly discriminate ALVR in the primary analyses. Although higher NETosis values were associated with ALVR in exploratory analyses using an ROC-derived threshold and correlated with selected baseline CMR parameters, these findings should be interpreted cautiously because they were secondary and hypothesis-generating. Prior STEMI studies have linked circulating NET-related markers with infarct size, ventricular dysfunction, adverse remodeling, and clinical outcomes after PCI [13,22], suggesting that NETosis may reflect the severity of acute myocardial injury rather than serve as a robust independent predictor of later remodeling. By contrast, direct human evidence supporting circulating 15(S)-HETE specifically as a predictor of post-STEMI remodeling remains limited. Available data provide greater support for altered eicosanoid pathway activity and possible fibrosis-related mechanistic involvement than for established clinical prognostic utility [21,23,24]. Overall, these findings indicate limited prognostic utility of single early measurements of 15(S)-HETE and NETosis activity in this context.

### 3.1. Clinical Implications

Among the biomarkers evaluated, platelet count measured early after PCI was the most readily accessible marker associated with subsequent ALVR, though its discriminatory performance was only moderate and not sufficient to support its use as a stand-alone clinical tool. At the same time, the observed association of lower 20-HETE with ALVR is biologically interesting, as it supports the potential relevance of eicosanoid-related pathways in post-infarction remodeling.

From a precision medicine perspective, these findings support the idea that combining easily accessible clinical markers with pathway-specific biomarkers could enhance risk stratification after STEMI. However, the current data are still insufficient to justify routine biomarker-guided management. Further research involving serial biomarker measurements, larger populations, and integration with genetic and molecular data is necessary to better understand how these pathways might help in developing personalized treatment strategies.

### 3.2. Limitations

This study has several limitations. First, it was a single-center observational study with a modest sample size, and only 19 patients developed ALVR. Although the multivariable models were pre-specified and intentionally restricted to reduce overfitting, the number of ALVR events was limited and the events-per-variable ratio remained low. The regression estimates should therefore be interpreted with caution, and our findings should be confirmed in larger cohorts. Second, only patients with paired baseline and 6-month follow-up CMR were included in the final analysis, which may have introduced selection bias and may limit generalizability to broader STEMI populations, particularly to patients with more severe clinical status, contraindications to CMR, or incomplete follow-up. Third, biomarker measurements were obtained at a single post-PCI time point, precluding assessment of temporal trajectories and limiting insight into the dynamic relationship between these biomarkers and remodeling. This is particularly relevant for dynamic biomarkers such as NETosis activity and eicosanoids, whose circulating levels may vary considerably during the acute post-infarction and reperfusion phases as part of the evolving inflammatory response. Therefore, differences in sampling timing relative to symptom onset and reperfusion may have introduced biological variability and attenuated the observed associations. Although most patients underwent primary PCI, a small subset received thrombolysis before PCI; therefore, heterogeneity in reperfusion strategy may have contributed to variability in early biomarker levels. Residual confounding is also likely, particularly because established markers of infarct severity and baseline ventricular injury may overlap with the observed biomarker associations. Fourth, the primary outcome was an imaging surrogate defined by ALVR on follow-up CMR rather than a major clinical outcome, such as heart failure, hospitalization, or mortality. In addition, the 6-month follow-up period may not capture later remodeling patterns or long-term prognostic implications. Finally, analyses of ROC-derived cut-off values, LVEF improvement, and correlations were exploratory, were not adjusted for multiple comparisons, and were not externally validated; these findings should therefore be considered hypothesis-generating.

## 4. Materials and Methods

### 4.1. Study Design and Population

**Figure 2:**
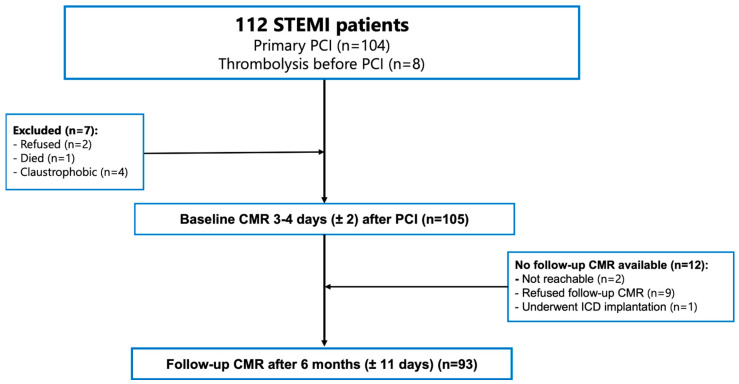
Study flow diagram.

Of the 112 initially enrolled patients, 7 were excluded before baseline imaging. Among the remaining 105 patients, follow-up CMR at 6 months was unavailable in 12, leaving 93 with paired baseline and follow-up CMR data for the final analysis. The diagnosis of STEMI was established according to the Fourth Universal Definition of Myocardial Infarction, based on characteristic ischemic symptoms, ST-segment elevation on electrocardiography (ECG), elevated cardiac troponin I levels (upper reference limit 0.04 µg/L), and angiographic documentation of an acutely occluded coronary artery [25]. All patients received reperfusion therapy (including primary PCI in 104 patients and thrombolysis followed by PCI in 8 patients) within 24 h of symptom onset and received guideline-directed medical therapy in accordance with contemporary European Society of Cardiology recommendations [26]. Baseline CMR was performed at a median of 4 days (IQR, 3–6) after PCI, and follow-up CMR was scheduled for 6 months later (±11 days). Patient flow is shown in Figure 2.

### 4.2. Eligibility Criteria and Clinical Data

Eligible participants were patients aged >18 years with a first STEMI treated with reperfusion therapy within 24 h of symptom onset. Patients were excluded if they had a history of ischemic heart disease, including previous myocardial infarction, previous PCI, or coronary artery bypass grafting; moderate or severe valvular heart disease, including prior valve surgery; previous myocarditis; active malignancy; estimated glomerular filtration rate < 30 mL/min/1.73 m^2^; or pregnancy. Additional exclusion criteria were standard contraindications to CMR, such as incompatible ferromagnetic implants or devices, intracranial aneurysm clips, or severe claustrophobia. Patients with atrial fibrillation at the time of CMR acquisition were also excluded because of the potential impact on image quality and strain analysis. Clinical data were extracted from electronic medical records and included age, sex, comorbidities, cardiovascular risk factors, pre-admission medications, coronary angiography findings, PCI procedural details, and in-hospital complications of STEMI. Anthropometric measurements, including weight and height, were recorded. Body surface area was calculated using the Du Bois formula: 0.007184 × weight (kg)^0.425^ × height (cm)^0.725^. Body mass index was calculated as weight (kg) divided by height squared (m^2^).

### 4.3. Ethics

The study was conducted in accordance with the Declaration of Helsinki and was approved by the Kaunas Regional Biomedical Research Ethics Committee (Approval No. BE-2-6, approved 5 February 2020). All patients provided written informed consent before enrollment.

### 4.4. Blood Sampling and Biomarker Assays

Fasting venous blood samples were collected on the morning after PCI. Routine laboratory parameters were measured in the central laboratory of the Hospital of the Lithuanian University of Health Sciences Kauno klinikos, Kaunas, Lithuania, using standard institutional methods. Biomarker analyses were performed at the Laboratory of Molecular Cardiology, Institute of Cardiology, Lithuanian University of Health Sciences, Kaunas, Lithuania.

Plasma samples were used to measure 20-HETE and 15(S)-HETE. Eicosanoids were isolated from plasma by organic extraction. Samples were transferred to sealed microcentrifuge tubes, supplemented with butylated hydroxytoluene (10 mg/mL), and acidified with acetic acid (>99%) to approximately pH 4. Extraction was performed with ethyl acetate (1:1, *v*/*v*). Samples were vortex-mixed for 2 min and centrifuged at 2000 rpm for 10 min at room temperature. The upper organic phase was collected and evaporated to dryness under a stream of nitrogen at 40 °C using a Multivap Nitrogen Evaporator (Organomation Associates, Berlin, MA, USA). The dried residue was reconstituted in 20 μL ethanol and further diluted with 130 μL of 1× sample dilution buffer before analysis. Concentrations of 20-HETE and 15(S)-HETE were determined using commercially available competitive enzyme-linked immunosorbent assay kits (Abcam, Cambridge, UK; Cat# ab175817 and ab133035, respectively). Optical density was measured using an Infinite M Plex microplate reader (Tecan, Männedorf, Switzerland) at 450 nm for 20-HETE and at 405 nm with a reference wavelength of 590 nm for 15(S)-HETE.

NETosis activity was assessed in serum using a colorimetric neutrophil elastase assay kit (Abcam, UK; Cat# ab235979). Serum samples were diluted 1:10 with assay buffer, incubated with neutrophil elastase substrate in a 96-well plate for 1.5 h at 37 °C, and absorbance was measured at 405 nm using the same reader.

### 4.5. Cardiac Magnetic Resonance Imaging Acquisition

All CMR examinations were performed on a 3.0-T clinical scanner (Magnetom Skyra, Siemens Healthineers, Erlangen, Germany) using an 18-channel phased-array body coil in combination with a spine coil. A standardized imaging protocol was applied at baseline and at 6-month follow-up. LV cine images were acquired using a balanced steady-state free precession sequence with retrospective ECG gating during end-expiratory breath-holds. Imaging parameters were as follows: echo time 1.39 ms, repetition time 2.5 ms, field of view 320–360 mm, matrix 192 × 146, flip angle 47°, slice thickness 8 mm, and interslice gap 2 mm. Contiguous short-axis cine slices covering the entire left ventricle from base to apex were obtained, together with standard long-axis views (two-chamber, three-chamber, and four-chamber). These cine acquisitions were used for assessment of LV volumes, left ventricular ejection fraction (LVEF)**,** and myocardial deformation parameters. Image acquisition was performed in accordance with standardized Society for Cardiovascular Magnetic Resonance recommendations for CMR imaging after myocardial infarction [27].

### 4.6. Image Analysis

CMR images were analyzed offline by a single experienced reader (AV, with >5 years of CMR experience) using the commercially available Medis Suite software (version 3.2, Medis Medical Imaging Systems, Leiden, The Netherlands). The reader was blinded to clinical data and outcomes at the time of image analysis. Analysis followed the Society for Cardiovascular Magnetic Resonance standardized guidelines for LV function and mass quantification [28].

Left ventricular end-diastolic volume (LVEDV) and end-systolic volume (LVESV) were quantified by tracing endocardial and epicardial contours on short-axis cine images at end-diastole and end-systole, respectively, with papillary muscles and trabeculations included in the LV cavity (blood pool method). LVEF, LV stroke volume, and LV myocardial mass were derived accordingly. All volumetric parameters were indexed to body surface area. Volumetric analysis was performed using QMass.

Myocardial deformation (strain) analysis was performed using feature tracking with QStrain. Long-axis cine images (two-chamber, three-chamber, and four-chamber views) were used to assess global longitudinal strain (GLS), while short-axis cine images (basal, mid-ventricular, and apical slices) were used to assess global circumferential strain (GCS). Endocardial and epicardial contours were defined at end-diastole and automatically propagated throughout the cardiac cycle using the software’s tracking algorithm, with manual adjustments applied when necessary to ensure accurate tracking. GLS and GCS were calculated as the average of peak segmental strain values according to the American Heart Association 16-segment model [29].

### 4.7. Definition of Outcomes

The primary outcome was adverse left ventricular remodeling (ALVR), defined as a ≥12% increase in both LVEDV and LVESV at 6-month follow-up compared with baseline. This CMR-specific threshold, proposed by Bulluck et al. [4], was selected to capture measurable adverse changes in LV volumes. The combined LVEDV and LVESV criterion was used because an increase in both volumes indicates a more consistent pattern of adverse ventricular enlargement than a change in either volume alone. This definition has also been associated with adverse long-term clinical outcomes in a STEMI CMR cohort.

### 4.8. Statistical Analysis

All analyses were performed in patients with paired baseline and 6-month CMR data (*n* = 93). Continuous variables are presented as median with IQR, and categorical variables as counts and percentages. Between-group comparisons were performed using the Mann–Whitney U test for continuous variables and the chi-square test or Fisher’s exact test, as appropriate. Changes in CMR parameters from baseline to 6-month follow-up were assessed using the Wilcoxon signed-rank test. Correlations between laboratory or biomarker variables and CMR-derived parameters were evaluated using Spearman’s rank correlation. Univariable and multivariable binary logistic regression analyses were performed to examine factors associated with adverse left ventricular remodeling (ALVR). The main multivariable model was specified a priori and included 20-HETE, urea, and platelet count. With 19 events and 3 covariates, the events-per-variable ratio was approximately 6.3, which is below the traditional rule-of-thumb of ≥10. To limit the risk of overfitting, the model was pre-specified, restricted to 3 clinically and biologically relevant variables, and no stepwise selection was used. Receiver operating characteristic (ROC) analysis was used to assess discrimination for ALVR, with area under the curve (AUC) and 95% confidence intervals (CIs) reported. Optimal cut-off values were determined using the Youden index and were used to dichotomize continuous variables for exploratory logistic regression analyses. Additional exploratory analyses examined factors associated with an absolute improvement in LVEF ≥ 10% at 6-month follow-up using logistic regression, with a limited number of variables included due to the small number of events. Analyses of LVEF improvement, ROC-derived thresholds, and correlations were considered exploratory and hypothesis-generating, and no adjustment for multiple comparisons was performed. All tests were two-sided, with *p* < 0.05 considered statistically significant. Analyses were performed using IBM SPSS Statistics version 31.0 (IBM Corp., Armonk, NY, USA).

## 5. Conclusions

In patients with a first STEMI treated with reperfusion therapy, about 20% developed adverse left ventricular remodeling despite overall improvement in LV systolic function during follow-up. Lower platelet count early after PCI was independently associated with later ALVR and may represent a simple, clinically accessible marker of increased risk of unfavorable post-infarction remodeling. Additionally, lower 20-HETE levels were associated with ALVR and showed moderate discriminatory ability, suggesting a possible role for eicosanoid pathways in post-infarction remodeling. This finding should, however, be considered exploratory given the small sample size and borderline statistical significance. Neither 15(S)-HETE nor NETosis activity showed a consistent association with ALVR in the main analyses. Overall, these findings suggest that combining accessible clinical biomarkers with pathway-specific indicators may improve risk stratification after STEMI, although larger and mechanistically focused studies are needed for validation.

## Figures and Tables

**Table 1:** Baseline clinical, procedural, and laboratory characteristics according to ALVR status in STEMI patients with paired CMR data (*n* = 93).

Characteristic	Overall (*n* = 93)	No ALVR (*n* = 74)	ALVR (*n* = 19)	*p*-Value
**Demographics and cardiovascular risk factors**				
Age, years	61.0 (55.0–67.5)	61.0 (53.0–67.3)	61.0 (57.0–71.0)	0.407
Male sex, *n* (%)	72 (77.4)	56 (75.7)	16 (84.2)	0.548
BMI, kg/m^2^	28.1 (25.0–30.8)	28.0 (24.7–30.5)	28.4 (25.8–32.4)	0.214
Systolic blood pressure, mmHg	143.0 (129.0–160.0)	145.0 (129.5–159.0)	138.0 (128.0–164.0)	0.699
Diastolic blood pressure, mmHg	84.0 (76.0–91.0)	84.5 (76.0–92.3)	82.0 (74.0–89.0)	0.298
Family history of CAD, *n* (%)	34 (36.6)	29 (39.2)	5 (26.3)	0.299
Diabetes mellitus, *n* (%)	12 (12.9)	9 (12.2)	3 (15.8)	0.705
Hypertension, *n* (%)	76 (81.7)	59 (79.7)	17 (89.5)	0.509
Dyslipidemia, *n* (%)	85 (91.4)	68 (91.9)	17 (89.5)	0.664
Active smoking, *n* (%)	48 (51.6)	36 (48.6)	12 (63.2)	0.259
**Pre-hospital medications**				
Beta-blockers, *n* (%)	30 (32.3)	20 (27.0)	10 (52.6)	0.033 *
ACE inhibitors or ARBs, *n* (%)	44 (47.3)	31 (41.9)	13 (68.4)	0.039 *
Calcium channel blockers, *n* (%)	11 (11.8)	6 (8.1)	5 (26.3)	0.028 *
Thiazide/thiazide-like diuretics, *n* (%)	14 (15.1)	9 (12.2)	5 (26.3)	0.124
Statins, *n* (%)	8 (8.6)	7 (9.5)	1 (5.3)	0.561
Antiplatelet drugs, *n* (%)	2 (2.2)	1 (1.4)	1 (5.3)	0.294
**Clinical and procedural characteristics**				
Onset-to-balloon time, min	345.0 (197.5–532.5)	364.0 (203.8–576.3)	275.0 (170.0–440.0)	0.341
Thrombolysis before PCI, *n* (%)	6 (6.5)	4 (5.4)	2 (10.5)	0.598
Anterior MI, *n* (%)	38 (40.9)	26 (35.1)	12 (63.2)	0.027 *
Pre-PCI TIMI flow 0–1, *n* (%)	67 (72.0)	54 (73.0)	13 (68.4)	0.922
Post-PCI TIMI flow 3, *n* (%)	87 (93.5)	68 (91.9)	19 (100.0)	0.439
**Markers of myocardial injury and complications**				
Killip class ≥ 2, *n* (%)	22 (23.7)	17 (23.0)	5 (26.3)	0.767
**Laboratory and biomarker parameters**				
Platelet count, ×10^9^/L	217.0 (188.0–242.0)	222.0 (196.5–245.8)	191.0 (181.0–233.0)	0.022 *
Troponin I, µg/L	26.6 (14.2–45.7)	26.6 (11.2–47.9)	25.4 (17.7–41.1)	0.523
BNP, ng/L	154.8 (94.2–248.9)	154.8 (93.5–245.1)	157.1 (94.8–319.3)	0.407
Creatinine, µmol/L	71.0 (59.8–86.0)	71.0 (59.3–80.5)	76.5 (59.8–94.5)	0.360
eGFR, mL/min/1.73 m^2^	95.0 (82.2–101.0)	95.0 (85.5–101.6)	89.0 (78.0–100.0)	0.177
Urea, mmol/L	5.2 (4.3–6.7)	5.2 (4.2–6.5)	6.1 (4.8–7.7)	0.067
20-HETE, ng/mL	23.7 (3.3–95.7)	37.1 (6.8–124.1)	6.2 (0.1–32.4)	0.004 *
15(S)-HETE, ng/mL	0.5 (0.2–1.4)	0.4 (0.2–1.4)	0.8 (0.5–1.7)	0.141
NETosis activity, mU/mL	54.1 (42.6–69.8)	54.1 (42.6–68.5)	57.5 (45.0–105.3)	0.257
**Baseline CMR parameters**				
LVEF, %	49.0 (40.7–52.4)	50.2 (41.0–52.9)	45.0 (39.4–49.3)	0.107
LVESV, mL	91.8 (75.1–113.8)	89.0 (70.5–110.1)	102.1 (91.5–133.4)	0.029 *
LVEDV, mL	177.2 (149.5–210.0)	175.6 (148.3–201.4)	197.8 (164.1–224.0)	0.092

Values are presented as median (interquartile range) or *n* (%), as appropriate. * *p* < 0.05. Pre-hospital medications refer to treatment used before hospitalization. 20-HETE = 20-hydroxyeicosatetraenoic acid; 15(S)-HETE = 15(S)-hydroxyeicosatetraenoic acid; ACE = angiotensin-converting enzyme; ALVR = adverse left ventricular remodeling; ARB = angiotensin receptor blocker; BMI = body mass index; BNP = B-type natriuretic peptide; CAD = coronary artery disease; CMR = cardiac magnetic resonance; eGFR = estimated glomerular filtration rate; LVEDV = left ventricular end-diastolic volume; LVEF = left ventricular ejection fraction; LVESV = left ventricular end-systolic volume; MI = myocardial infarction; PCI = percutaneous coronary intervention; TIMI = Thrombolysis in Myocardial Infarction.

**Table 2:** Changes in CMR parameters from baseline to 6-month follow-up in patients with STEMI (*n* = 93).

Parameter	Baseline	6-Month Follow-Up	*p*-Value
LVEF, %	49.0 (40.7–52.4)	55.8 (47.9–59.9)	<0.001 *
LVESV, mL	91.8 (75.1–113.8)	80.8 (61.9–108.3)	0.005 *
LVEDV, mL	177.2 (149.5–210.0)	178.3 (146.5–214.5)	0.274
LV GLS, %	−20.6 (−23.0–−17.6)	−23.9 (−26.8–−20.5)	<0.001 *
LV GCS, %	−27.2 (−31.3–−23.4)	−31.0 (−35.9–−26.2)	<0.001 *

Values are presented as median (interquartile range) (*n* = 93). * *p* < 0.05. CMR = cardiac magnetic resonance; LV = left ventricle; LVEF = left ventricular ejection fraction; LVESV = left ventricular end-systolic volume; LVEDV = left ventricular end-diastolic volume; GLS = global longitudinal strain; GCS = global circumferential strain.

**Table 3:** Univariable and multivariable logistic regression analysis for predictors of ALVR (*n* = 93).

Variable	Univariable OR (95% CI)	*p*-Value	Multivariable OR (95% CI)	*p*-Value
Age, years	1.03 (0.98–1.09)	0.275	—	—
20-HETE, ng/mL	0.98 (0.96–1.00)	0.091	0.99 (0.97–1.00)	0.047 *
Urea, mmol/L	1.34 (1.02–1.78)	0.039 *	1.38 (1.00–1.91)	0.051
Platelet count, ×10^9^/L	0.99 (0.97–1.00)	0.029 *	0.98 (0.97–1.00)	0.015 *

ORs are presented with 95% CIs. * *p* < 0.05. ALVR = adverse left ventricular remodeling; OR = odds ratio; CI = confidence interval.

**Table 4:** Diagnostic performance of biomarkers for prediction of ALVR.

Variable	AUC (95% CI)	*p*-Value	Cut-Off	Sensitivity (%)	Specificity (%)
20-HETE	0.713 (0.59–0.83)	<0.001 *	39.75	94.7	48.6
15(S)-HETE	0.612 (0.49–0.74)	0.083	0.45	77.8	54.1
NETosis activity	0.585 (0.43–0.74)	0.271	95.25	31.6	94.6
Platelet count	0.670 (0.55–0.79)	0.007 *	209.0	68.4	66.2

Data are presented as AUCs with 95% CIs. Optimal cut-off values were determined using the Youden index. Sensitivity and specificity are reported for the selected cut-off values. * *p* < 0.05. For 20-HETE and platelet count, lower values were associated with ALVR. ALVR = adverse left ventricular remodeling; AUC = area under the curve; CI = confidence interval.

**Table 5:** Univariable logistic regression analysis of ROC-derived biomarkers for prediction of ALVR.

Variable	ROC-Derived Category	OR (95% CI)	*p*-Value
20-HETE	≤39.75 vs. >39.75	17.05 (2.16–134.41)	0.007 *
15(S)-HETE	≥0.45 vs. <0.45	4.12 (1.24–13.69)	0.021 *
NETosis activity	≥95.25 vs. <95.25	8.08 (2.00–32.65)	0.003 *
Platelet count	≤209.0 vs. >209.0	4.25 (1.44–12.51)	0.009 *
BNP	≥291.15 vs. <291.15	5.42 (1.61–18.26)	0.006 *
Urea	≥6.05 vs. <6.05	2.80 (1.00–7.88)	0.050 *

ROC-derived cut-off values were determined using the Youden index. ORs are presented for the category associated with a higher risk of ALVR relative to the reference category. * *p* < 0.05. ALVR = adverse left ventricular remodeling; BNP = B-type natriuretic peptide; CI = confidence interval; OR = odds ratio; ROC = receiver operating characteristic.

**Table 6:** Multivariable logistic regression analysis of ROC-derived predictors of ALVR.

Variable	ROC-Derived Category	Adjusted OR (95% CI)	*p*-Value
20-HETE	≤39.75 vs. >39.75	29.55 (2.80–311.53)	0.005 *
BNP	≥291.15 vs. <291.15	8.07 (1.60–40.87)	0.012 *
Platelet count	≤209.0 vs. >209.0	4.93 (1.25–19.50)	0.023 *
NETosis activity	≥95.25 vs. <95.25	16.87 (2.38–119.32)	0.005 *

Adjusted ORs are presented with 95% CIs. Variables were entered simultaneously into the multivariable binary logistic regression model and dichotomized according to the ROC-derived cut-off values shown in Table 5. No relevant collinearity was observed among included variables. * *p* < 0.05. ALVR = adverse left ventricular remodeling; OR = odds ratio; CI = confidence interval; BNP = B-type natriuretic peptide.

## Data Availability

The data presented in this study are available on request from the corresponding author. The data are not publicly available due to privacy and ethical restrictions related to patient confidentiality and the conditions of the ethics committee approval.

## Supplementary Materials

The following supporting information can be downloaded at: https://www.mdpi.com/article/10.3390/ph19050794/s1, Table S1: Clinical, laboratory, and imaging variables ac-cording to improvement in LVEF at 6-month follow-up (*n* = 93); Table S2: Univariable and multi-variable logistic regression for prediction of improvement in LVEF at 6-month follow-up (*n* = 93); Table S3: Spearman correlations between LV function/remodeling parameters and laboratory/biomarker variables; Table S4: Correlations of biomarkers with LV myocardial deformation parameters.

## Abbreviations

20-HETE	20-hydroxyeicosatetraenoic acid
15(S)-HETE	15(S)-hydroxyeicosatetraenoic acid
ALVR	adverse left ventricular remodeling
AUC	area under the curve
CI	confidence interval
CMR	cardiac magnetic resonance
ECG	electrocardiography
GCS	global circumferential strain
GLS	global longitudinal strain
HDL	high-density lipoprotein
LV	left ventricular
LVEDV	left ventricular end-diastolic volume
LVEF	left ventricular ejection fraction
LVESV	left ventricular end-systolic volume
NETs	neutrophil extracellular traps
PCI	percutaneous coronary intervention
ROC	receiver operating characteristic
STEMI	ST-segment elevation myocardial infarction
