# Supplementary material for: Clinical and Biomarker Predictors of Adverse Left Ventricular Remodeling After First STEMI: Insights into Phenotype Variability Using CMR
Source: Pharmaceuticals (Basel). 2026 May 19;19(5):794. doi: 10.3390/ph19050794 (PMC13209677; doi:10.3390/ph19050794)
Supplement: Supplementary file 1 [file pharmaceuticals-19-00794-s001.zip › pharmaceuticals-4294010-supplementary.pdf]

Supplementary material

Clinical and Biomarker Predictors of Adverse Left Ventricular Remodeling After First STEMI: Insights into Phenotype Variability Using CMR

Agneta Virbickiene [1,2,*], Vacis Tatarunas [1], Ieva Ciapiene [1], Neda Jonaitiene [2], Justina Jureviciute[2], Paulius Bucius [2], Arnoldas Leleika [2], Ieva Jonauskiene [4], Liepa Kleizaite [3], Tomas Lapinskas [2] and Olivija Dobiliene [2]

Table S1. Clinical, laboratory, and imaging variables according to improvement in LVEF at 6-month follow-up (n = 93)

Variable	LVEF improvement <10% (n = 71)	LVEF improvement ≥10% (n = 22)	p-value
Age, years	61.0 (54.0–68.5)	63.0 (57.5–65.0)	0.597
Onset-to-balloon time, min	330 (197–532)	390 (150–671)	0.606
Baseline LVEF, %	50.7 (41.8–54.7)	42.3 (40.0–48.0)	**0.004***
Troponin I, μg/L	29.6 (13.4–53.5)	26.1 (15.1–40.2)	0.563
20-HETE, ng/mL	35.3 (4.2–121.6)	9.6 (2.8–37.8)	0.120
15(S)-HETE, ng/mL	0.50 (0.20–1.50)	0.40 (0.20–1.30)	0.478
NETosis activity, mU/mL	56.1 (42.6–74.6)	47.6 (36.3–64.7)	0.164
BNP, ng/L	150.0 (88.8–242.5)	210.2 (117.5–300.3)	0.065
HDL, mmol/L	1.16 (0.98–1.36)	1.30 (1.17–1.48)	**0.029***
Urea, mmol/L	5.50 (4.60–6.75)	4.30 (3.75–5.60)	**0.006***
Family history of CAD, n (%)	22 (31.0)	12 (54.5)	**0.041***
Anterior MI, n (%)	23 (32.4)	15 (68.2)	**0.003***

Values are presented as median (interquartile range) or n (%), as appropriate. Continuous variables were compared using the Mann-Whitney U test, and categorical variables using the chi-square test or Fisher's exact test, as appropriate. Improvement in LVEF was defined as an absolute increase of ≥10% between baseline and 6-month follow-up. * $p < 0.05$. Analyses were exploratory, and no adjustment for multiple comparisons was performed.

BNP = B-type natriuretic peptide; CAD = coronary artery disease; HDL = high-density lipoprotein; LVEF = left ventricular ejection fraction; MI = myocardial infarction.

Table S2. Univariable and multivariable logistic regression for prediction of improvement in LVEF at 6-month follow-up (n = 93)

Baseline predictor	Univariable OR (95% CI)	p-value	Multivariable OR (95% CI)	p-value
Family history of CAD	2.67 (1.01–7.11)	**0.049***	—	—
Urea, mmol/L	0.67 (0.47–0.95)	**0.026***	—	—
HDL, mmol/L	5.93 (1.16–30.25)	**0.032***	7.84 (1.26–48.99)	**0.028***

Binary logistic regression was used to identify predictors of improvement in LVEF at 6-month follow-up in patients with paired CMR data. Improvement in LVEF was defined as an absolute increase of ≥10% between baseline and follow-up. Variables with $p < 0.10$ in univariable analysis were considered for multivariable analysis. ORs are presented with 95% CIs. * $p < 0.05$. Analyses were exploratory and hypothesis-generating; no adjustment for multiple comparisons was performed.

CAD = coronary artery disease; CI = confidence interval; LVEF = left ventricular ejection fraction; OR = odds ratio.

Table S3. Spearman correlations between LV function/remodeling parameters and laboratory/biomarker variables

	Baseline						Follow-up					
Variable	LVEF		LVESV		LVEDV		LVEF		LVESV		LVEDV	
	r	p	r	p	r	p	r	p	r	p	r	p
20-HETE, ng/mL	0.145	0.143	-0.057	0.568	0.005	0.963	0.096	0.360	0.024	0.820	0.147	0.160
NETosis activity, mU/mL	-0.038	0.703	0.196	**0.047***	0.263	**0.007***	-0.078	0.458	0.159	0.129	0.164	0.116
BNP, ng/L	-0.313	**0.001***	0.054	0.593	-0.129	0.198	-0.223	**0.033***	0.042	0.691	-0.082	0.437
Platelet count, $\times 10^9$/L	0.188	0.056	-0.220	**0.025***	-0.180	0.068	0.229	**0.027***	-0.189	0.070	-0.125	0.232
Troponin I, μg/L	-0.539	**<0.001***	0.454	**<0.001***	0.314	**0.001**	-0.594	**<0.001***	0.527	**<0.001***	0.376	**<0.001***
ALT, IU/L	-0.412	**<0.001***	0.316	**0.001***	0.188	0.056	-0.439	**<0.001***	0.360	**<0.001***	0.231	**0.026***
AST, IU/L	-0.544	**<0.001***	0.398	**<0.001***	0.217	**0.027***	-0.576	**<0.001***	0.454	**<0.001***	0.294	**0.004***

Values are presented as Spearman correlation coefficients (r) with corresponding p-values. Correlations of laboratory and biomarker variables with LV functional and remodeling parameters at baseline and 6-month follow-up were assessed using Spearman's rank correlation. * $p < 0.05$. Analyses were exploratory and hypothesis-generating; no adjustment for multiple comparisons was performed.

ALT = alanine aminotransferase; AST = aspartate aminotransferase; BNP = B-type natriuretic peptide; HETE = hydroxyeicosatetraenoic acid; LV = left ventricle; LVEDV = left ventricular end-diastolic volume; LVEF = left ventricular ejection fraction; LVESV = left ventricular end-systolic volume.

Table S4. Correlations of biomarkers with LV myocardial deformation parameters

	Baseline				Follow-up			
**Variable**	**GLS r**	**GLS p**	**GCS r**	**GCS p**	**GLS r**	**GLS p**	**GCS r**	**GCS p**
20-HETE, ng/mL	**-0.217**	**0.027***	-0.027	0.788	-0.003	0.980	-0.007	0.949
NETosis activity, mU/mL	**0.210**	**0.032***	0.149	0.131	0.132	0.211	0.082	0.437
BNP, ng/L	**0.213**	**0.033***	**0.272**	**0.006***	0.135	0.205	0.181	0.087
Platelet count, $\times 10^9$/L	-0.133	0.179	-0.190	0.054	0.013	0.900	-0.124	0.237
Troponin I, µg/L	**0.470**	**<0.001***	**0.507**	**<0.001***	**0.521**	**<0.001***	**0.518**	**<0.001***
ALT, IU/L	**0.375**	**<0.001***	**0.373**	**<0.001***	**0.288**	**0.005***	**0.402**	**<0.001***
AST, IU/L	**0.467**	**<0.001***	**0.483**	**<0.001***	**0.431**	**<0.001***	**0.444**	**<0.001***

Values are presented as Spearman correlation coefficients (r) with corresponding p-values. Correlations of laboratory and biomarker variables with LV myocardial deformation parameters at baseline and 6-month follow-up were assessed using Spearman's rank correlation. * $p < 0.05$. Analyses were exploratory and hypothesis-generating; no adjustment for multiple comparisons was performed.

ALT = alanine aminotransferase; AST = aspartate aminotransferase; BNP = B-type natriuretic peptide; GCS = global circumferential strain; GLS = global longitudinal strain; HETE = hydroxyeicosatetraenoic acid; LV = left ventricle.
